# Absence of dopaminergic neuronal degeneration and oxidative damage in aged *DJ-1*-deficient mice

**DOI:** 10.1186/1750-1326-2-10

**Published:** 2007-05-29

**Authors:** Hiroo Yamaguchi, Jie Shen

**Affiliations:** 1 Center for Neurologic Diseases, Brigham & Women's Hospital, Program in Neuroscience, Harvard Medical School, Boston, MA, 02115, USA

## Abstract

Parkinson's disease is the most common movement disorder characterized by dopaminergic dysfunction and degeneration. Loss-of-function mutations in the *DJ-1 *gene have been linked to autosomal recessive forms of early-onset familial Parkinson's disease. DJ-1 is thought to play roles in protection of cells against oxidative stress and in maintenance of the normal dopaminergic function in the nigrostriatal pathway. Here we investigate the consequence of both DJ-1 inactivation and aging in mice. We found that *DJ-1*-/- mice at the age of 24–27 months have normal numbers of dopaminergic neurons in the substantia nigra and normal levels of dopamine and its major metabolites in the striatum. The number of noradrenergic neurons in the locus coeruleus is also unchanged in *DJ-1*-/- mice. Moreover, there is no accumulation of oxidative damage or inclusion bodies in aged *DJ-1*-/- brains. Together, these results indicate that loss of DJ-1 function alone is insufficient to cause nigral degeneration and oxidative damage in the life span of mice.

## Background

Parkinson's disease (PD) is an age-related movement disorder characterized clinically by bradykinesia, rigidity, resting tremor and postural instability, and neuropathologically by the selective loss of dopaminergic (DA) neurons and the presence of Lewy bodies in the substantia nigra (SN). Although most PD cases are sporadic, mutations in *parkin *(PARK2), *PINK1 *(PARK6), and *DJ-1 *(PARK7) have been linked to recessively inherited forms of parkinsonism, which resemble idiopathic PD clinically [1-3]. To investigate how DJ-1 deficiency causes PD, we have previously generated a mouse model bearing a targeted germline disruption of *DJ-1*, and our multidisciplinary analysis has uncovered an essential role for DJ-1 in DA physiology and dopamine D2 receptor-mediated functions [4].

Besides the importance of DJ-1 in DA neurotransmission and signaling, DJ-1 has been reported to have multiple functions associated with PD pathogenesis. First, several cysteine residues in DJ-1 can be oxidized in response to oxidative stress, and wild-type but not mutant DJ-1 protects cells from oxidative stress [5-11]. Furthermore, DJ-1 has been shown to stabilize the antioxidant transcription master regulator Nrf2 (nuclear factor erythroid 2-related factor) [12]. Second, DJ-1 has chaperone activity and inhibits α-synuclein aggregation, which is thought to be a key event in Lewy body formation [13]. Third, it has been suggested that DJ-1 might be involved in transcriptional regulation of neuroprotective or anti-apoptotic genes [14].

DA neurons are likely to be exposed to increased levels of oxidative stress caused by the metabolic products of dopamine in comparison to other types of neurons in the brain. It is thought that reactive oxygen species (ROS) oxidizes lipids, proteins and nucleic acids, resulting in cellular dysfunction or death [15,16]. Evidence has shown that products of lipid, protein and DNA oxidation accumulate in PD brains [17,18]. It has been shown that levels of DJ-1 protein are significantly increased in PD brains and cerebrospinal fluids, and DJ-1 is oxidatively damaged in the brains of patients with sporadic PD [19-21]. Therefore, it has been hypothesized that DJ-1 plays a critical role in antioxidant mechanisms and preventing cellular dysfunction or death in DA neurons. Consistent with this notion, DJ-1 deficiency induces an increased sensitivity to oxidative stimuli, including hydrogen peroxide, 6-hydroxydopamine, and 1-methyl-4-phenyl 1,2,3,6-tetrahydropyridine (MPTP), and overexpression DJ-1 protects neurons from various oxidative stimuli [5,10,22-25]. Furthermore, Drosophila *DJ-1 *mutants showed accumulation of ROS and are sensitive to oxidative stress including paraquat, rotenone or hydrogen peroxide [9,26]. *DJ-1*-/- mice showed increased sensitivity to MPTP and oxidative stress [23]. It however remains unclear whether DJ-1 deficiency would lead to accumulation of oxidative damage in aging mouse brains in the absence of environmental oxidative stressors.

## Results

**Figure 1:**
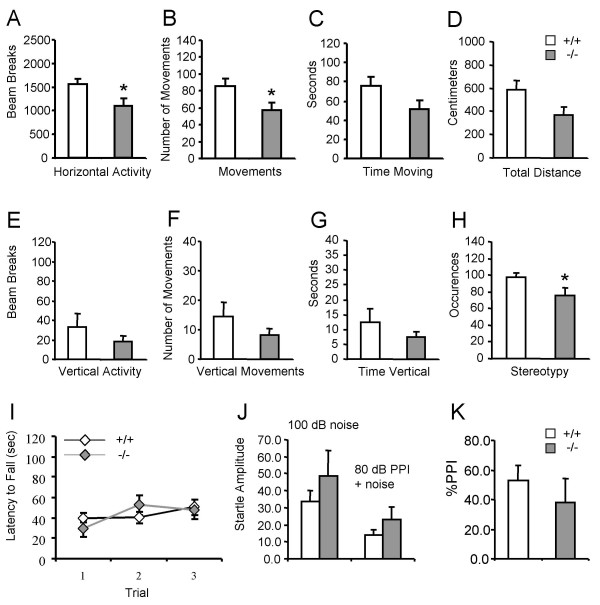
**Aged *DJ-1 *-/- mice exhibit reduced spontaneous activity in the open field. (A**-**H) **Evaluation of *DJ-1*-/- mice and wild-type controls at the age of 18–25 months (+/+: n = 17, -/-: n = 15) in the open field for 15 min. Two arrays measured horizontal movements (A-D), and one array measured vertical movements (rearing on hind legs) (E-G). Repeated sequential breakings of the same beam are scored as occurrences of stereotyped behaviors (scratching, grooming, etc.) (H). *DJ-1*-/- mice show significantly reduced horizontal activity, horizontal movements and stereotypy (p < 0.05). **(I) ***DJ-1*-/- and wild-type control mice at the age of 22–25 months show similar latencies to fall off an accelerating rotating rod during 3 trials (+/+: n = 9, -/-: n = 8, p > 0.05). **(J **and **K) ***DJ-1*-/- and wild-type mice at the age of 22–25 months show similar acoustic startle responses to 100 dB noise alone (+/+: 33.5 ± 6.7, n = 9, -/-: 48.5 ± 15.1, n = 6, p > 0.05), 100 dB noise with 80 dB PPI (+/+: 13.7 ± 3.1, n = 9, -/-: 22.8 ± 7.5, n = 6, p > 0.05) and %PPI (+/+: 52.9 ± 9.9, n = 9, -/-: 38.2 ± 16.1, n = 6, p > 0.05). Data in all panels are expressed as mean ± SEM. Asterisk denotes statistical significance (*p < 0.05).

Our previous study showed that *DJ-1*-/- mice displayed hypoactivity in the open field at the age of 3 months [4]. To examine whether aged *DJ-1*-/- mice display reduced locomotor activity, we assessed the locomotor abilities of aged *DJ-1*-/- mice using a battery of well-established behavioral tests. Recording of spontaneous, voluntary movements during 15 min in the open field test revealed a significant reduction in the horizontal activity and fewer instances of stereotyped behavior of *DJ-1*-/- mice (n = 15) compared to wild-type littermates (n = 17) at the age of 18–25 months (Fig. 1A–H). We also assessed involuntary movement using the rotarod and acoustic startle reflex paradigms. Rotarod test revealed that *DJ-1*-/- (n = 8) and wild-type littermate (n = 9) mice at the age of 22–25 months had similar latency before falling off the rotating rod during three independent trials (Fig. 1I). *DJ-1*-/- (n = 6) and control (n = 9) mice at the age of 22–25 months displayed similar acoustic startle reflex, measured by the force with which the mouse jumped in response to a pulse of loud noise (Fig. 1J). Acoustic startle response can be inhibited by a preceding weaker stimulus, a process termed prepulse inhibition (PPI), which is thought to be modulated by the central noradrenergic neurotransmission [27-29]. Aged DJ-1-/- mice displayed also normal PPI (Fig. 1K).

**Figure 2:**
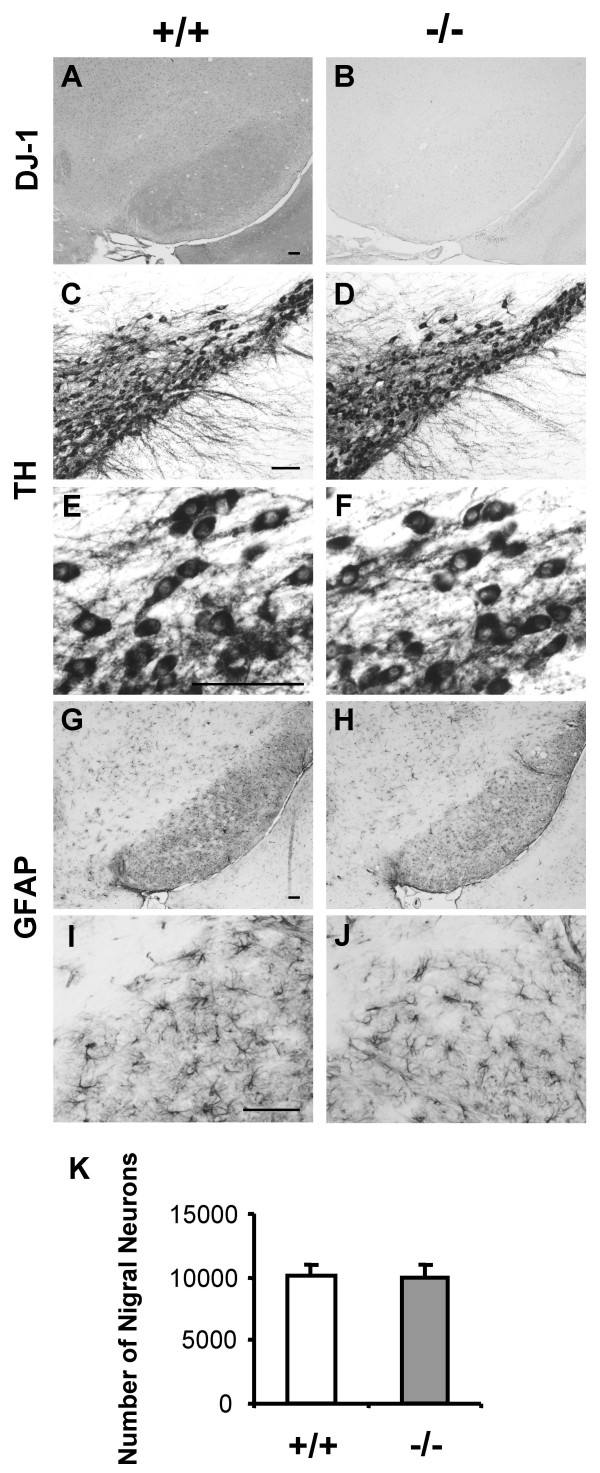
**No DA neuron loss in the SN in aged *DJ-1 *-/- mice. (A and B) **The lack of the expression of DJ-1 protein in the SN in *DJ-1*-/- mice is indicated by the presence of DJ-1 immunoreactivity in the SN of wild-type controls and the absence of DJ-1 immunoreactivity in *DJ-1*-/- mice. **(C-F) **Normal morphology of DA neurons in aged *DJ-1*-/- mice is indicated by similar TH staining in the SN of *DJ-1*-/- mice and wild-type controls at the age of 24–27 months. Panels (E, F) indicate enlarged view of panels (C, D), respectively.**(G-J) **Similar GFAP staining in the SN of *DJ-1*-/- mice and wild-type controls suggesting that there is no inflammatory or neurodegenerative changes in the SN of *DJ-1*-/- mice. Panels (I, J) indicate enlarged view of panels (G, H), respectively. Scale bars; A-J, 0.1 mm.**(K) **Similar numbers of TH-positive neurons are present in the SN of *DJ-1*-/- and wild-type mice at the age of 24–27 months (+/+: 10140 ± 812, -/-: 9960 ± 972, n = 4 per genotype, p > 0.05). All data are expressed as mean ± SEM.

To investigate the consequence of DJ-1 inactivation and aging on the survival of DA neurons, we performed quantitative histological analysis on *DJ-1*-/- mice at the age of 24–27 months. Immunohistochemical analysis of *DJ-1*-/- mice using an antibody against DJ-1 confirmed the absence of DJ-1 protein in the brain of *DJ-1*-/- mice (Fig. 2A, B). Nissl staining revealed normal brain morphology in aged *DJ-1*-/- mice (data not shown). Though the most prominent neuropathological feature of PD is the selective loss of DA neurons in the substantia nigra (SN), immunohistochemical analysis of aged *DJ-1*-/- mice using an antibody specific for tyrosine hydroxylase (TH) revealed normal TH staining in the SN and normal morphology of DA neurons at the age of 24–27 months (Fig. 2C–F). Quantification of the number of DA neurons in the SN of *DJ-1*-/- (n = 4) and control mice (n = 4) using unbiased stereological methods revealed similar numbers of TH-positive neurons in the SN of *DJ-1*-/- and wild-type mice (+/+: 10140 ± 812, -/-: 9960 ± 972, n = 4 per genotype, p > 0.05) (Fig. 2K). Since DJ-1 protein is also present in astrocytes [30,31], and increased glial fibrillary acidic protein (GFAP) immunoreactivity is a good marker for inflammatory responses and neurodegeneration, we also performed GFAP immunostaining. No difference in GFAP immunoreactivity and morphology of astrocytes was detected in the SN between *DJ-1*-/- and wild-type mice (Fig. 2G–J). These findings indicate that there is no DA neuronal degeneration in *DJ-1*-/- mice during the life span of mice.

**Figure 3:**
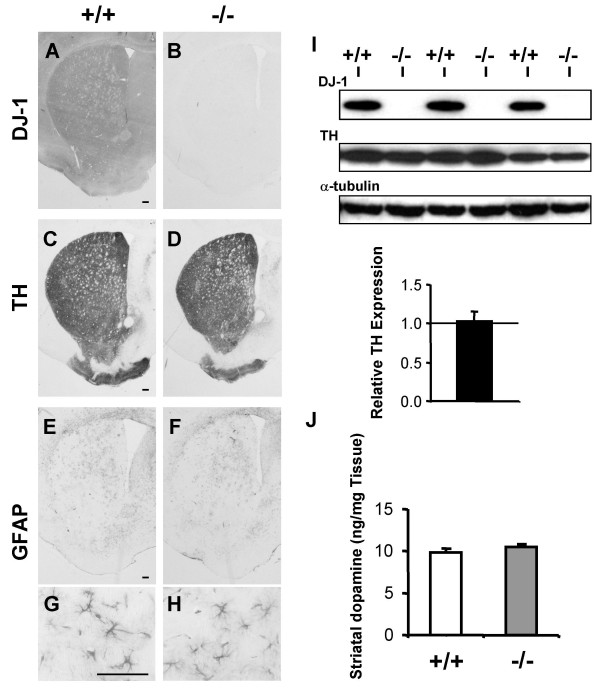
**No DA neuron terminal loss and normal dopamine content in the striatum in aged *DJ-1 *-/- mice. (A and B) **The absence of the expression of DJ-1 protein in the striatum in *DJ-1*-/- mice is indicated by the presence of DJ-1 immunoreactivity in the striatum of wild-type controls and the absence of the immunoreactivity in *DJ-1*-/- mice. **(C and D) **Similar TH staining in the striatum of *DJ-1*-/- mice and wild-type controls at the age of 18–19 months indicating no DA neuron terminal loss in *DJ-1*-/- mice. **(E-H) **Similar GFAP staining in the striatum of *DJ-1*-/- mice and wild-type controls suggesting that there is no inflammatory or neurodegenerative changes in *DJ-1*-/- mice. Panels (G, H) indicate enlarged view of panels (E, F), respectively. Scale bars; A-H, 0.1 mm.**(I) **Western analysis of DJ-1, TH, and α-tubulin proteins in the striatum of aged *DJ-1*-/- brains and wild-type controls at the age of 18–20 months. The value is normalized to that of α-tubulin. The level of TH proteins in brains of wild-type mice is set at 1.00 (TH: -/-; 1.03 ± 0.12, p > 0.05) (n = 4 per genotype). **(J) **Similar striatal content of dopamine in *DJ-1*-/- mice and wild-type controls at the age of 24–27 months (+/+: 9.8 ± 0.6, n = 7, -/-: 10.5 ± 0.4, n = 5, p > 0.05). All data are expressed as mean ± SEM.

Since DA neurons in the SN project processes to the striatum, we next examined the striatum. Immunohistochemical studies confirmed the absence of DJ-1 protein in the striatum of *DJ-1*-/- mice (Fig. 3A, B) and there was no significant difference in TH staining of DA nerve terminals in the striatum (Fig. 3C, D). Furthermore, there was no difference in GFAP immunoreactivity in the striatum between *DJ-1*-/- and control mice (Fig. 3E–H). Western analysis also showed unchanged levels of TH protein in the *DJ-1*-/- brain (Fig. 3I). HPLC analysis revealed that striatal levels of dopamine and its major metabolites, dihydroxyphenylacetic acid (DOPAC) and homovanillic acid (HVA) are similar between *DJ-1*-/- and control mice at the age of 24–27 months (Fig. 3J) (dopamine: +/+; 9.8 ± 0.6 ng/mg, n = 7, -/-; 10.5 ± 0.4 ng/mg, n = 5, p > 0.05; DOPAC: +/+; 2.0 ± 0.1 ng/mg, n = 7, -/-; 2.0 ± 0.2 ng/mg, n = 5, p > 0.05; HVA: +/+; 1.4 ± 0.2 ng/mg, n = 7, -/-; 1.2 ± 0.1 ng/mg, n = 5, p > 0.05). These findings indicate that levels of striatal dopamine are unchanged in *DJ-1*-/- mice during the life span of mice.

Lewy bodies are protein aggregates containing α-synuclein and ubiquitin and are considered a pathological hallmark of PD. Therefore, we examined *DJ-1*-/- brains for deposits of α-synuclein and ubiquitin. Immunohistochemical analysis of *DJ-1*-/- brains using antibodies specific for α-synuclein and ubiquitin showed no inclusions in any brain sub-regions, including the SN at the age of 24–27 months (data not shown).

**Figure 4:**
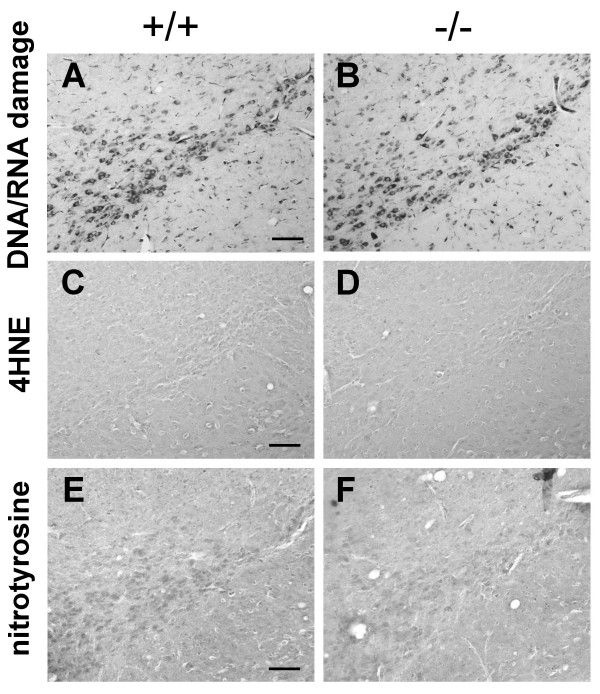
**No increased oxidative damage in the brains of *DJ-1 *-/- mice. (A and B) **Similar staining of DNA and RNA oxidative damage in the SN of *DJ-1*-/- mice and wild-type controls at the age of 24–27 months showing no abnormal accumulation of DNA and RNA oxidative damage in aged *DJ-1*-/- mice. **(C and D) **Similar 4HNE staining in the SN of *DJ-1*-/- mice and wild-type controls showing no abnormal accumulation of lipid peroxidation products in aged *DJ-1*-/- mice. **(E and F) **Similar nitrotyrosine staining in the SN of *DJ-1*-/- mice and wild-type controls showing no abnormal accumulation of oxidative protein damage in aged *DJ-1*-/- mice. Scale bars; A-F, 0.1 mm.

Oxidative damage is thought to contribute to the degeneration of DA neurons in PD [17]. DJ-1 is thought to play a role in anti-oxidative stress by scavenging ROS. It has been reported that oxidative DNA or RNA damage, such as 8-oxoguanine, accumulates in the SN in both PD patients and mouse models [32,33]. Several lipid peroxides, especially 4-hydroxy-2-nonenal (4HNE), are highly reactive, avidly form adducts with many proteins and have been detected in Lewy bodies [34]. Lewy bodies are also immunoreactive for 3-nitrotyrosine, an index of protein damage by ROS [35]. Immunohistochemical analysis of *DJ-1*-/- brains using these oxidative stress markers revealed there was no significant increase in these immunoreactivities in the SN at the age of 24–27 months (Fig. 4A–F), indicating that there is no accumulation of oxidative damage in aged *DJ-1*-/- brains.

**Figure 5:**
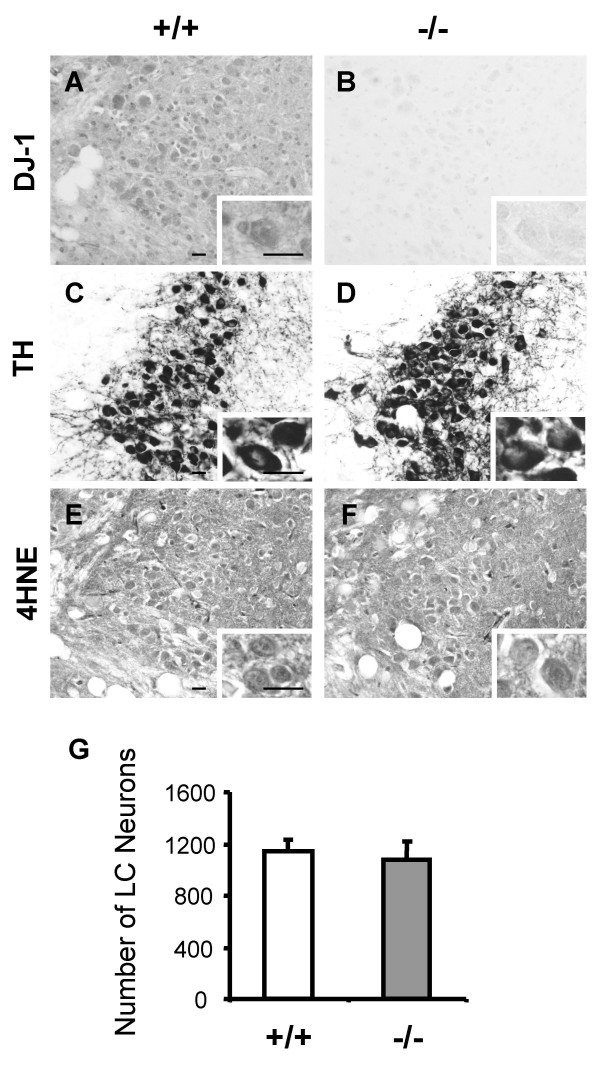
**Normal morphology of noradrenergic neuron and no noradrenergic neuron loss in LC in aged *DJ-1 *-/- mice. (A and B) **A substantial level of expression of DJ-1 in LC in control mice is indicated by the presence of DJ-1 immunoreactivity in the LC of wild-type controls and the absence of the immunoreactivity in *DJ-1*-/- mice. Any immunoreactivity in *DJ-1*-/- mice indicates non-specific staining. **(C and D) **Similar TH staining in the LC of *DJ-1*-/- mice and wild-type controls showing normal morphology of noradrenergic neurons in LC in aged *DJ-1*-/- mice at the age of 24–27 months. **(E and F) **Similar 4HNE staining in the LC of *DJ-1*-/- mice and wild-type controls showing no abnormal accumulation of lipid peroxidation products in aged *DJ-1*-/- mice. Insets in panels indicate enlarged view of each section. Scale bars; A-F, 0.02 mm; insets, 0.02 mm. **(G) **The total numbers of TH-positive LC neurons of both sides in all sections from rostral to caudal showing similar numbers of TH-positive neurons in the LC of *DJ-1*-/- mice and wild-type controls at the age of 24–27 months (+/+: 1145 ± 87, -/-: 1073 ± 151, p > 0.05) (n = 8 per genotype). All data are expressed as mean ± SEM.

In addition to DA degeneration, brains of PD patients also show degeneration of noradrenergic neurons in the locus coeruleus (LC). It was reported that *parkin*-deficient mice have a loss of catecholaminergic neurons in the LC [36]. Therefore, we examined whether aged *DJ-1*-/- mice would exhibit loss of noradrenergic neurons in the LC. Immunohistochemical analysis showed substantial levels of DJ-1 in the LC of wild-type mice (Fig. 5A, B). TH staining revealed normal morphology of noradrenergic neurons in the LC of *DJ-1*-/- mice at the age of 24–27 months (Fig. 5C, D). Immunohistochemical analysis using antibodies specific for α-synuclein and ubiquitin showed no inclusions in noradrenergic neurons (data not shown). 4HNE staining revealed that there was no significant difference in the immunoreactivity between *DJ-1*-/- mice and wild-type controls (Fig. 5E, F). The TH-positive noradrenergic neurons were counted in sections spanning the rostral-caudal extent of the nucleus at the age of 24–27 months. There was no significant difference in the total number of TH-positive noradrenergic neurons in the LC between *DJ-1*-/- mice and wild-type controls (+/+: 1145 ± 87, -/-: 1073 ± 151, p > 0.05; n = 8 per genotype; Fig. 5G). These findings indicate that there is no noradrenergic neuron loss in the LC of aged *DJ-1*-/- mice.

## Discussion

Mutations in *parkin *(PARK2), *PINK1 *(PARK6), and *DJ-1 *(PARK7) are associated with autosomal recessive PD, in which loss of function of each of these gene products leads to degeneration of DA neurons and clinical manifestations of PD. We previously reported that *DJ-1*-/- mice display significant motor abnormalities and nigrostriatal DA functional deficits, though the number and morphology of DA neurons are normal up to the age of 12 months [4]. Since aging is a major risk factor for PD, we analyzed older *DJ-1*-/- mice to determine whether aged *DJ-1*-/- mice developed PD-like pathology, such as degeneration of nigrostriatal DA neurons in the SN or noradrenergic neurons in the LC. Our quantitative analysis failed to detect any significant loss of DA neurons or noradrenergic neurons in aged *DJ-1*-/- mice at 24–27 months. We found that levels of striatal dopamine and its metabolites were normal. Additionally, there were no other neuropathological changes such as gliosis or protein aggregation in aged *DJ-1*-/- brains. Furthermore, we found no accumulation of oxidative damage in aged *DJ-1*-/- brains.

Despite the fact that multiple important functions associated with the pathogenesis of PD have been attributed to DJ-1, surprisingly, we found that loss of DJ-1 function in mice even at the age of 2 years did not cause significant loss of DA neurons. First, DJ-1 has been reported to function as an anti-oxidative stress agent through scavenging ROS [5-7]. However, we failed to find increases in immunoreactivities of oxidative damage markers in *DJ-1*-/- brains at the age of 24–27 months, suggesting the lack of accumulation of ROS in aged *DJ-1*-/- brains. It has been reported that expression of DJ-1 is induced in cells that have been subjected to oxidative stresses [5]. Therefore, it is possible that DJ-1 plays a critical role in an environment with elevated oxidative stress; however, under normal conditions, DJ-1 is not required for nigral neuron survival. To examine whether *DJ-1*-/- mice have increased susceptibility to oxidative stress under oxidative conditions is an important question to be addressed in future studies. Second, it was reported that DJ-1 had chaperone activity and inhibited α-synuclein aggregation [13]. Immunohistochemical studies in aged *DJ-1*-/- mice did not show any inclusions immunoreactive for α-synuclein or ubiquitin, indicating that loss of DJ-1 function is not enough to result in formation of these protein inclusions. It has been reported that these inclusions have been found in animal models treated with oxidative stimuli such as rotenone or MPTP [37,38] and that the chaperone activity of DJ-1 can be stimulated by oxidation [13]. Therefore, investigation of whether DJ-1 inactivation would accelerate protein aggregation under conditions of oxidative stimuli is necessary to understand the role of DJ-1 in chaperone activity and formation of Lewy bodies. Third, it has been suggested that DJ-1 might be involved in transcriptional regulation. DJ-1 transcriptionally up-regulates human TH by inhibiting the sumoylation of pyrimidine tract-binding protein-associated splicing factor (PSF) [39]. We however failed to detect reduced TH expression in *DJ-1*-/- mice even at the age of 2 years indicating that DJ-1 is not required in the transcriptional regulation of TH expression in mice.

In summary, despite the fact that loss of function mutations in DJ-1 cause PD and presumably nigral degeneration in humans, our current study failed to find DA neurodegeneration in *DJ-1*-/- mice during the life span of mice. In addition, although DJ-1 has been shown to protect cells from environmental oxidative stimuli, absence of DJ-1 did not cause accumulation of oxidative damage in aged *DJ-1*-/- mice under normal conditions. These results are consistent with our prior report showing that loss of parkin function alone in mice is also insufficient to cause loss of DA neurons up to the age of 2 years [40]. Other possibilities, including shorter life span of mice, well-controlled mouse housing environment, may contribute to the absence of profound nigral degeneration that is characteristic of PD brains.

## Methods

### Behavioral tests

*Open field: *Male *DJ-1*-/- mice and wild-type littermates were tested in the open field using two acrylic animal cages. Each pair of both genotypic groups were placed into two cages at a time for 15 min during which their horizontal and vertical movements were monitored using 3 arrays of 16 infrared light beam sensors (AccuScan Instruments). The total number of movements, the distance traveled, the time spent moving and the total number of infrared beam breaks in both the horizontal plane and along the vertical axis were recorded and analyzed using AccuScan VersaMax software. Statistical differences between the two genotypes were assessed by Student's *t*-test. *Rotarod: *Male *DJ-1*-/- mice and wild-type littermates were also tested on the rotarod. Two pairs of both genotypic groups were placed at one time on the Economex accelerating rotarod (Columbus Instruments) equipped with individual timers for each mouse. Mice were initially trained to stay on the rod for 2 min at a constant rotation speed of 5 rpm. After a 2 min rest, mice were returned to the rotating rod at an accelerating speed of 0.2 rpm/sec, and the time of the mice remaining on the rotating rod was measured as latency to fall. A total of 3 trials were performed for each mouse. *Acoustic Startle Reflex*: Noise and prepulse generation were controlled by a computer. Each pair of both genotypic groups were placed in the two calibrated startle cylinders (Med Associates) and received a 5-min acclimation period without background noise before the startle stimuli. The testing session contained 50 trials and lasted 25 minutes, which consisted of twenty five pulses at 100 dB alone or twenty five 100 dB pulses preceded (100 ms) by prepulses of 80 dB in a semi-random order with a 30-second interval. The stimulation duration was 600 ms, while the duration of 100 dB pulses was 50 ms at frequencies of 5–40 kHz and the duration of the prepulses at 80 dB was 10 ms at a frequency of 10 kHz. Their responses were measured with a transducer that was attached to the underside of the platform and connected to the computer. Averages of peak values resulting from 100 dB pulses alone or 100 dB pulses coupled with 80 dB prepulses were calculated. %PPI was calculated using the following formula; 100 – (startle amplitude with PPI/startle amplitude alone) × 100. The data was evaluated with Student's *t*-test.

### Histology and neuron counting

Mouse brains were dissected, formalin fixed for 2 h, processed for paraffin embedding, and sectioned in the coronal plane at 16 μm or 20 μm thickness. Each paraffin block contained 4 *DJ-1*-/- and 4 wild-type brains. Deparaffinized sections were immersed in a solution of 3% H_2_O_2_/methanol for 15 min. The sections were incubated in 10% normal goat serum (NGS)/phosphate buffered saline (PBS) for 1 h, and then were incubated with each appropriately diluted primary antibodies against DJ-1 (rabbit polyclonal; Signet), tyrosine hydroxylase (TH) (rabbit polyclonal; Chemicon), glial fibrillary acidic protein (GFAP) (mouse monoclonal; Sigma), α-synuclein (Syn-1; mouse monoclonal; BD Transduction Labs.), ubiquitin (rabbit polyclonal, DAKO), Michael adducts of 4HNE (rabbit polyclonal, Calbiochem), nitrotyrosine (rabbit polyclonal, Upstate) or DNA/RNA oxidative damage (mouse monoclonal, QED Bioscience) in 10% NGS/PBS at 4°C overnight. Rinsed sections were processed by Vectastain ABC kit (Vector Labs.) with the correct biotinylated secondary antibody, and the peroxidase reaction product was detected using DAB peroxidase substrate (Vector Labs.). The number of DA neurons in the SN was determined by counting TH immunoreactive neurons in coronal sections of four brains per genotype using the fractionator and optical dissector methods of unbiased stereology [41] under a Leica DMRB microscope equipped with a CCD camera connected to a computer running Bioquant image analysis software. The counting of the number of TH-positive cells in LC was performed by counting the cells in every 4 coronal sections (16 μm thickness) from the rostralmost to caudalmost limits of the LC [42,43]. A TH-positive cell was defined as an immunoreactive somata with a clearly visible unstained nucleus, or a piece of a soma of comparable size. The cells were counted bilaterally in all sections per animal with a power (200X) using a light microscope. The total number of TH-positive LC neurons per animal was calculated by summing the bilateral TH-positive LC neurons in all sections from rostral to caudal. The experimenter was blind to the genotypes of mice. Values are reported as means ± SEM. Statistical differences were assessed by Student's t test.

### Striatal dopamine and metabolites measurements by HPLC

Striata were dissected, weighed and stored at -80°C. Frozen striata were sonicated in ice-cold solution (0.1 N perchloric acid, 0.2 mM sodium bisulfite) and centrifuged for 20 min at 20,000 × g at 4°C. For dopamine measurement, the supernatant was filtered (0.2 μm) and applied to a C18 reverse phase HPLC column connected to an ESA model 5200A electrochemical detector with a 5014B microdialysis cell with potentials set to -175 mV and +200 mV using MD-TM mobile phase (ESA, Inc.) with isocratic elution. For metabolites measurement, the supernatant was applied to a 150 × 2.1 mm ID, C18 reverse phase HPLC column connected to an Alexys LC-100 system (Antec-Leyden) with electrochemical detection (DECADE II) and a VT-03 electrochemical flow cell using a detection potential of 590 mV and isocratic elution (50 mM phosphoric acid, 50 mM citric acid, 400 mg/ml OSA, 0.1 mM EDTA, 8 mM KCl, pH 3.75, 3% methanol) flowing at 0.2 ml/min.

### Western blotting

The dorsal striata were dissected out and sonicated in 500 μL of 150 mM NaCl, 50 mM Tris, pH 7.4, 2 mM EDTA, 1% Nonidet P-40, 1% sodiumdeoxycholate, 1% sodium dodecylsulfate, protease inhibitors (Roche) and phosphatase inhibitors (Calbiochem). The protein content was analyzed by BCA assay (Pierce), and 10 μg of protein per lane was resolved on 4–12% gradient gels (Invitrogen), transferred to nitrocellulose membrane, blocked with 5% milk in TBST (50 mM Tris, pH 7.4, 150 mM NaCl, 0.1% Tween-20), and incubated with a primary antibody (TH, Chemicon) at 4°C overnight. The membrane was then incubated with a peroxidase-conjugated anti-rabbit antibody (Biorad), treated with chemiluminescence reagent (PerkinElmer Life Sciences) and exposed to film. Sample was reprobed with a primary antibody against α-tubulin (mouse monoclonal; Sigma) to confirm equal protein loading.

## Abbreviations

DA, dopaminergic; DOPAC, 3,4-dihydroxyphenylacetic acid; GFAP, glial fibrillary acidic protein; 4HNE, 4-hydroxy-2-nonenal; HPLC, high performance liquid chromatography; HVA, homovanillic acid; LC, locus coeruleus; MPTP, 1-methyl-4-phenyl-1,2,3,6-tetrahydropyridine; PD, Parkinson's disease; PPI, prepulse inhibition; ROS, reactive oxygen species; SN, substantia nigra; TH, tyrosine hydroxylase

## Competing interests

The author(s) declare that they have no competing interests.

## Authors' contributions

HY and JS designed the experiments and wrote the paper; HY performed all of the experiments. All authors read and approved the final manuscript.

## References

[B1] Kitada T, Asakawa S, Hattori N, Matsumine H, Yamamura Y, Minoshima S, Yokochi M, Mizuno Y, Shimizu N (1998). Mutations in the parkin gene cause autosomal recessive juvenile parkinsonism. Nature.

[B2] Bonifati V, Rizzu P, van Baren MJ, Schaap O, Breedveld GJ, Krieger E, Dekker MC, Squitieri F, Ibanez P, Joosse M, van Dongen JW, Vanacore N, van Swieten JC, Brice A, Meco G, van Duijn CM, Oostra BA, Heutink P (2003). Mutations in the DJ-1 gene associated with autosomal recessive early-onset parkinsonism. Science.

[B3] Valente EM, Abou-Sleiman PM, Caputo V, Muqit MM, Harvey K, Gispert S, Ali Z, Del Turco D, Bentivoglio AR, Healy DG, Albanese A, Nussbaum R, Gonzalez-Maldonado R, Deller T, Salvi S, Cortelli P, Gilks WP, Latchman DS, Harvey RJ, Dallapiccola B, Auburger G, Wood NW (2004). Hereditary early-onset Parkinson's disease caused by mutations in PINK1. Science.

[B4] Goldberg MS, Pisani A, Haburcak M, Vortherms TA, Kitada T, Costa C, Tong Y, Martella G, Tscherter A, Martins A, Bernardi G, Roth BL, Pothos EN, Calabresi P, Shen J (2005). Nigrostriatal dopaminergic deficits and hypokinesia caused by inactivation of the familial Parkinsonism-linked gene DJ-1. Neuron.

[B5] Taira T, Saito Y, Niki T, Iguchi-Ariga SM, Takahashi K, Ariga H (2004). DJ-1 has a role in antioxidative stress to prevent cell death. EMBO Rep.

[B6] Canet-Aviles RM, Wilson MA, Miller DW, Ahmad R, McLendon C, Bandyopadhyay S, Baptista MJ, Ringe D, Petsko GA, Cookson MR (2004). The Parkinson's disease protein DJ-1 is neuroprotective due to cysteine-sulfinic acid-driven mitochondrial localization. Proc Natl Acad Sci U S A.

[B7] Kinumi T, Kimata J, Taira T, Ariga H, Niki E (2004). Cysteine-106 of DJ-1 is the most sensitive cysteine residue to hydrogen peroxide-mediated oxidation in vivo in human umbilical vein endothelial cells. Biochem Biophys Res Commun.

[B8] Menzies FM, Yenisetti SC, Min KT (2005). Roles of Drosophila DJ-1 in survival of dopaminergic neurons and oxidative stress. Curr Biol.

[B9] Meulener M, Whitworth AJ, Armstrong-Gold CE, Rizzu P, Heutink P, Wes PD, Pallanck LJ, Bonini NM (2005). Drosophila DJ-1 mutants are selectively sensitive to environmental toxins associated with Parkinson's disease. Curr Biol.

[B10] Martinat C, Shendelman S, Jonason A, Leete T, Beal MF, Yang L, Floss T, Abeliovich A (2004). Sensitivity to oxidative stress in DJ-1-deficient dopamine neurons: an ES- derived cell model of primary Parkinsonism. PLoS Biol.

[B11] Takahashi-Niki K, Niki T, Taira T, Iguchi-Ariga SM, Ariga H (2004). Reduced anti-oxidative stress activities of DJ-1 mutants found in Parkinson's disease patients. Biochem Biophys Res Commun.

[B12] Clements CM, McNally RS, Conti BJ, Mak TW, Ting JP (2006). DJ-1, a cancer- and Parkinson's disease-associated protein, stabilizes the antioxidant transcriptional master regulator Nrf2. Proc Natl Acad Sci U S A.

[B13] Shendelman S, Jonason A, Martinat C, Leete T, Abeliovich A (2004). DJ-1 is a redox-dependent molecular chaperone that inhibits alpha-synuclein aggregate formation. PLoS Biol.

[B14] Xu J, Zhong N, Wang H, Elias JE, Kim CY, Woldman I, Pifl C, Gygi SP, Geula C, Yankner BA (2005). The Parkinson's disease-associated DJ-1 protein is a transcriptional co-activator that protects against neuronal apoptosis. Hum Mol Genet.

[B15] Karam LR, Bergtold DS, Simic MG (1991). Biomarkers of OH radical damage in vivo. Free Radic Res Commun.

[B16] Halliwell B (1992). Reactive oxygen species and the central nervous system. J Neurochem.

[B17] Lotharius J, Brundin P (2002). Pathogenesis of Parkinson's disease: dopamine, vesicles and alpha-synuclein. Nat Rev Neurosci.

[B18] Nakabeppu Y, Tsuchimoto D, Yamaguchi H, Sakumi K (2007). Oxidative damage in nucleic acids and Parkinson's disease. J Neurosci Res.

[B19] Moore DJ, Zhang L, Troncoso J, Lee MK, Hattori N, Mizuno Y, Dawson TM, Dawson VL (2005). Association of DJ-1 and parkin mediated by pathogenic DJ-1 mutations and oxidative stress. Hum Mol Genet.

[B20] Waragai M, Wei J, Fujita M, Nakai M, Ho GJ, Masliah E, Akatsu H, Yamada T, Hashimoto M (2006). Increased level of DJ-1 in the cerebrospinal fluids of sporadic Parkinson's disease. Biochem Biophys Res Commun.

[B21] Choi J, Sullards MC, Olzmann JA, Rees HD, Weintraub ST, Bostwick DE, Gearing M, Levey AI, Chin LS, Li L (2006). Oxidative damage of DJ-1 is linked to sporadic Parkinson and Alzheimer diseases. J Biol Chem.

[B22] Yokota T, Sugawara K, Ito K, Takahashi R, Ariga H, Mizusawa H (2003). Down regulation of DJ-1 enhances cell death by oxidative stress, ER stress, and proteasome inhibition. Biochem Biophys Res Commun.

[B23] Kim RH, Smith PD, Aleyasin H, Hayley S, Mount MP, Pownall S, Wakeham A, You-Ten AJ, Kalia SK, Horne P, Westaway D, Lozano AM, Anisman H, Park DS, Mak TW (2005). Hypersensitivity of DJ-1-deficient mice to 1-methyl-4-phenyl-1,2,3,6-tetrahydropyrindine (MPTP) and oxidative stress. Proc Natl Acad Sci U S A.

[B24] Zhou W, Freed CR (2005). DJ-1 up-regulates glutathione synthesis during oxidative stress and inhibits A53T alpha-synuclein toxicity. J Biol Chem.

[B25] Inden M, Taira T, Kitamura Y, Yanagida T, Tsuchiya D, Takata K, Yanagisawa D, Nishimura K, Taniguchi T, Kiso Y, Yoshimoto K, Agatsuma T, Koide-Yoshida S, Iguchi-Ariga SM, Shimohama S, Ariga H (2006). PARK7 DJ-1 protects against degeneration of nigral dopaminergic neurons in Parkinson's disease rat model. Neurobiol Dis.

[B26] Yang Y, Gehrke S, Haque ME, Imai Y, Kosek J, Yang L, Beal MF, Nishimura I, Wakamatsu K, Ito S, Takahashi R, Lu B (2005). Inactivation of Drosophila DJ-1 leads to impairments of oxidative stress response and phosphatidylinositol 3-kinase/Akt signaling. Proc Natl Acad Sci U S A.

[B27] Bakshi VP, Geyer MA (1997). Phencyclidine-induced deficits in prepulse inhibition of startle are blocked by prazosin, an alpha-1 noradrenergic antagonist. J Pharmacol Exp Ther.

[B28] Saitoh K, Shaw S, Tilson HA (1986). Noradrenergic influence on the prepulse inhibition of acoustic startle. Toxicol Lett.

[B29] Toufexis DJ, Rochford J, Walker CD (1999). Lactation-induced reduction in rats' acoustic startle is associated with changes in noradrenergic neurotransmission. Behav Neurosci.

[B30] Kotaria N, Hinz U, Zechel S, von Bohlen Und Halbach O (2005). Localization of DJ-1 protein in the murine brain. Cell Tissue Res.

[B31] Bandopadhyay R, Kingsbury AE, Cookson MR, Reid AR, Evans IM, Hope AD, Pittman AM, Lashley T, Canet-Aviles R, Miller DW, McLendon C, Strand C, Leonard AJ, Abou-Sleiman PM, Healy DG, Ariga H, Wood NW, de Silva R, Revesz T, Hardy JA, Lees AJ (2004). The expression of DJ-1 (PARK7) in normal human CNS and idiopathic Parkinson's disease. Brain.

[B32] Shimura-Miura H, Hattori N, Kang D, Miyako K, Nakabeppu Y, Mizuno Y (1999). Increased 8-oxo-dGTPase in the mitochondria of substantia nigral neurons in Parkinson's disease. Ann Neurol.

[B33] Yamaguchi H, Kajitani K, Dan Y, Furuichi M, Ohno M, Sakumi K, Kang D, Nakabeppu Y (2006). MTH1, an oxidized purine nucleoside triphosphatase, protects the dopamine neurons from oxidative damage in nucleic acids caused by 1-methyl-4-phenyl-1,2,3,6-tetrahydropyridine. Cell Death Differ.

[B34] Castellani RJ, Perry G, Siedlak SL, Nunomura A, Shimohama S, Zhang J, Montine T, Sayre LM, Smith MA (2002). Hydroxynonenal adducts indicate a role for lipid peroxidation in neocortical and brainstem Lewy bodies in humans. Neurosci Lett.

[B35] Good PF, Hsu A, Werner P, Perl DP, Olanow CW (1998). Protein nitration in Parkinson's disease. J Neuropathol Exp Neurol.

[B36] Von Coelln R, Thomas B, Savitt JM, Lim KL, Sasaki M, Hess EJ, Dawson VL, Dawson TM (2004). Loss of locus coeruleus neurons and reduced startle in parkin null mice. Proc Natl Acad Sci U S A.

[B37] Betarbet R, Sherer TB, MacKenzie G, Garcia-Osuna M, Panov AV, Greenamyre JT (2000). Chronic systemic pesticide exposure reproduces features of Parkinson's disease. Nat Neurosci.

[B38] Fornai F, Schluter OM, Lenzi P, Gesi M, Ruffoli R, Ferrucci M, Lazzeri G, Busceti CL, Pontarelli F, Battaglia G, Pellegrini A, Nicoletti F, Ruggieri S, Paparelli A, Sudhof TC (2005). Parkinson-like syndrome induced by continuous MPTP infusion: convergent roles of the ubiquitin-proteasome system and alpha-synuclein. Proc Natl Acad Sci U S A.

[B39] Zhong N, Kim CY, Rizzu P, Geula C, Porter DR, Pothos EN, Squitieri F, Heutink P, Xu J (2006). DJ-1 transcriptionally up-regulates the human tyrosine hydroxylase by inhibiting the sumoylation of pyrimidine tract-binding protein-associated splicing factor. J Biol Chem.

[B40] Goldberg MS, Fleming SM, Palacino JJ, Cepeda C, Lam HA, Bhatnagar A, Meloni EG, Wu N, Ackerson LC, Klapstein GJ, Gajendiran M, Roth BL, Chesselet MF, Maidment NT, Levine MS, Shen J (2003). Parkin-deficient mice exhibit nigrostriatal deficits but not loss of dopaminergic neurons. J Biol Chem.

[B41] Sterio DC (1984). The unbiased estimation of number and sizes of arbitrary particles using the disector. J Microsc.

[B42] German DC, Liang CL, Manaye KF, Lane K, Sonsalla PK (2000). Pharmacological inactivation of the vesicular monoamine transporter can enhance 1-methyl-4-phenyl-1,2,3,6-tetrahydropyridine-induced neurodegeneration of midbrain dopaminergic neurons, but not locus coeruleus noradrenergic neurons. Neuroscience.

[B43] German DC, Nelson O, Liang F, Liang CL, Games D (2005). The PDAPP mouse model of Alzheimer's disease: locus coeruleus neuronal shrinkage. J Comp Neurol.

